# Somatic mutations in *CDH1* and *CTNNB1* in primary carcinomas at 13 anatomic sites

**DOI:** 10.18632/oncotarget.21115

**Published:** 2017-09-21

**Authors:** Evan L. Busch, Jason L. Hornick, Renato Umeton, Adem Albayrak, Neal I. Lindeman, Laura E. MacConaill, Elizabeth P. Garcia, Matthew Ducar, Timothy R. Rebbeck

**Affiliations:** ^1^ Channing Division of Network Medicine, Department of Medicine, Brigham and Women’s Hospital and Harvard Medical School, Boston, MA 02115, USA; ^2^ Department of Epidemiology, Harvard T.H. Chan School of Public Health, Boston, MA 02115, USA; ^3^ Department of Medical Oncology, Dana-Farber Cancer Institute, Boston, MA 02215, USA; ^4^ Department of Pathology, Brigham and Women’s Hospital and Harvard Medical School, Boston, MA 02115, USA; ^5^ Department of Informatics, Dana-Farber Cancer Institute, Boston, MA 02215, USA

**Keywords:** metastasis, carcinomas, epithelial-mesenchymal transition, mutation, detachment

## Abstract

Metastases are involved in most cancer deaths. Evidence has suggested that cancer cell detachment from primary tumors might occur largely via the mechanism of epithelial-mesenchymal transition (EMT) activated by epigenetic events, but data addressing other possible triggers of detachment, particularly genetic mutations, have been limited. Using the Profile study of cancer genomics at Dana-Farber Cancer Institute, we examined somatic mutations in the EMT genes *CDH1* in 5,106 primary carcinomas and *CTNNB1* in 7,578 primary carcinomas across 13 anatomic sites: urinary bladder, breast, colon/rectum, endometrium, esophagus, kidney, lung, ovary, pancreas, prostate, skin (non-melanoma), stomach, and thyroid. For each gene and anatomic site, we calculated the prevalence of primary carcinomas with at least one mutation. Across all anatomic sites, 4% of carcinomas had at least one *CDH1* mutation and 4% of carcinomas had at least one *CTNNB1* mutation. By anatomic site, the observed prevalence of carcinomas with at least one mutation was less than 5% at 10 sites for *CDH1* and 12 sites for *CTNNB1*. Tumor stage data were available for a subset of breast, colorectal, lung, and prostate tumors. Among patients from this subset who were diagnosed with regional or distant disease, only 4% had a *CDH1* mutation and 1% had a *CTNNB1* mutation in the primary tumor. The low mutation prevalences, especially among those with diagnoses of regional or distant disease, suggest that somatic mutations in *CDH1* and *CTNNB1* are unlikely to explain a substantial proportion of cancer cell detachment from primary carcinomas originating at most anatomic sites.

## INTRODUCTION

Metastases contribute to most cancer deaths [1]. Developing successful approaches to preventing and treating metastatic disease requires understanding how cancer cells detach from primary tumors and acquire the ability to migrate throughout the body.

Epithelial-mesenchymal transition (EMT) is an important mechanism of metastasis. When undergoing EMT, cancer cells temporarily reduce expression of epithelial markers and increase expression of mesenchymal markers [2, 3]. Reduced expression of epithelial markers, especially of the cell-cell adhesion molecule *CDH1* (E-cadherin), contributes to cancer cell detachment from the primary tumor [3]. Changes in expression of *CTNNB1* (beta-catenin), which binds to *CDH1*, are also associated with EMT [3]. A substantial body of research suggests that the expression changes in epithelial and mesenchymal markers that are observed in EMT are largely induced by epigenetic processes [4–6]. However, establishing epigenetics as the prime trigger of detachment also requires ruling out other possibilities, such as mutations in EMT genes. If epigenetic events are the main cause of detachment, one would expect mutations to be rare in genes whose expression levels are involved in detachment, but few studies to date have attempted to evaluate this. In addition, past studies of mutations in EMT genes have had either small sample sizes [7–10] or been difficult to interpret in terms of detachment due to not restricting their analyses to primary tumor specimens [11].

We used the Profile study of cancer genomics at Dana-Farber Cancer Institute (DFCI; Boston, MA) [12] to evaluate whether mutations in key EMT genes might contribute to the detachment of cancer cells from primary tumors. Specifically, we examined somatic mutations in *CDH1* and *CTNNB1* in thousands of clinical primary carcinomas from 13 anatomic sites. Based on earlier work suggesting that epigenetic mechanisms play a major role in EMT, we hypothesized that the prevalence of primary carcinomas with at least one mutation for either gene would be too low to explain the proportions of cancer cases that clinically manifest cancer cell detachment from primary tumors.

## RESULTS

**Figure 1:**
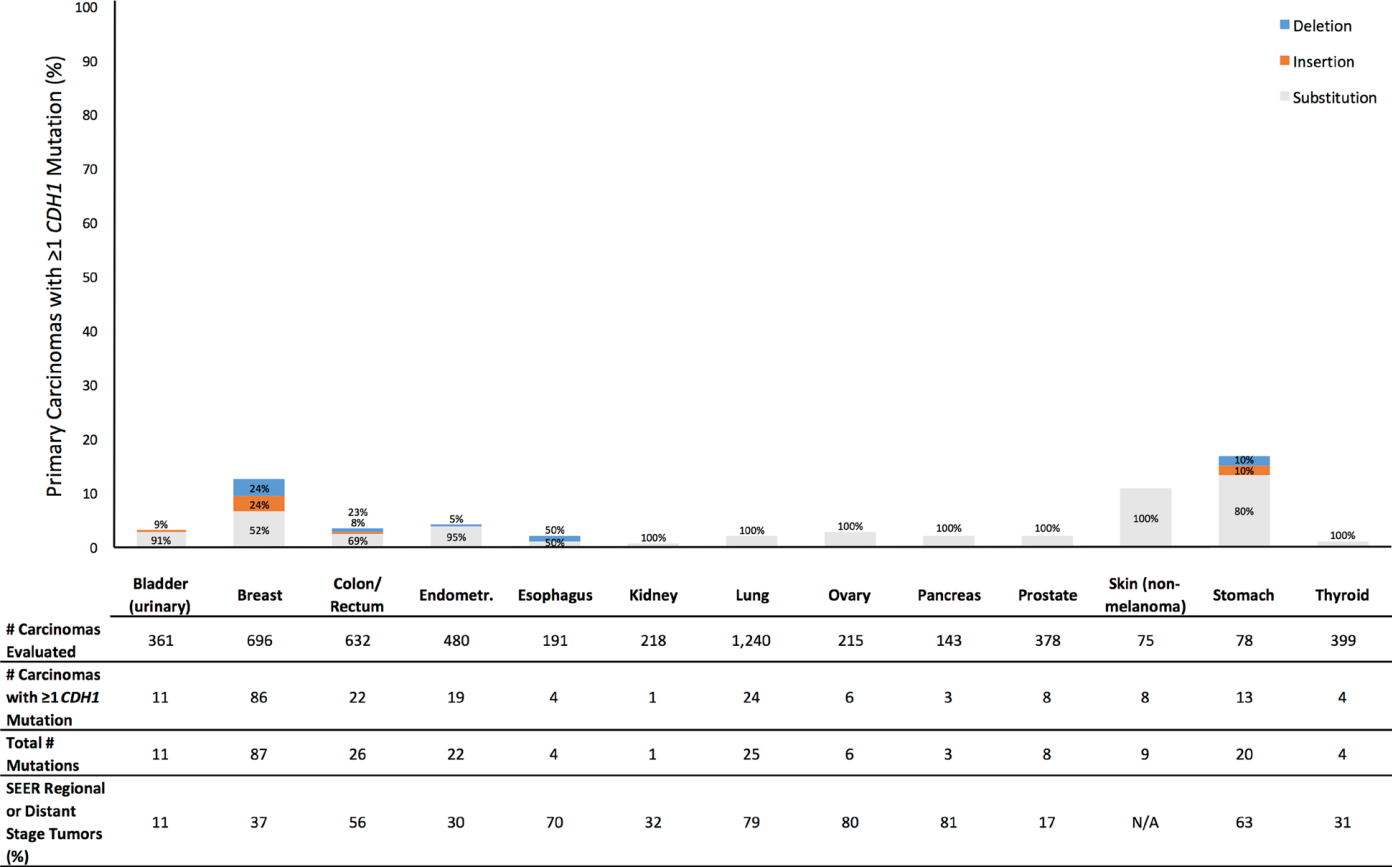
Prevalence of Profile primary carcinomas by anatomic site with at least one *CDH1* mutation (overall height of bar) and distribution of types of mutations (percentages within bar), number of primary carcinomas evaluated, total number of *CDH1* mutations observed across all evaluated tumors, and SEER percentage of cases diagnosed with tumor stage of regional or distant disease (SEER Source: SEER 18 2007–2013, All Races, by SEER Summary Stage 2000). Profile data and SEER data are based on different sets of tumors

**Figure 2:**
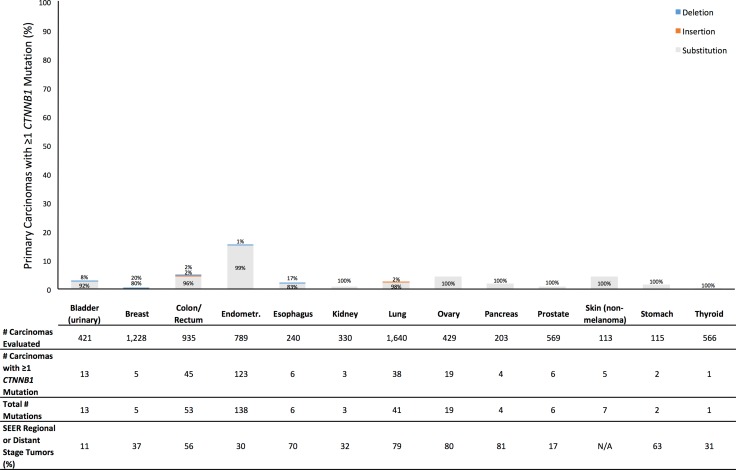
Prevalence of Profile primary carcinomas by anatomic site with at least one *CTNNB1* mutation (overall height of bar) and distribution of types of mutations (percentages within bar), number of primary carcinomas evaluated, total number of *CTNNB1* mutations observed across all evaluated tumors, and SEER percentage of cases diagnosed with tumor stage of regional or distant disease (SEER Source: SEER 18 2007–2013, All Races, by SEER Summary Stage 2000). Profile data and SEER data are based on different sets of tumors

Across the 13 anatomic sites, 5,106 eligible primary carcinomas were evaluated for *CDH1* and 7,578 eligible primary carcinomas were evaluated for *CTNNB1*. All carcinomas evaluated for *CDH1* were also evaluated for *CTNNB1*. For each anatomic site, the number of carcinomas evaluated for *CDH1* is given in Figure 1 and the number of carcinomas evaluated for *CTNNB1* is given in Figure 2. The maximum sample size by site ranged from 113 carcinomas (non-melanoma skin) to 1,640 (lung) (Figure 2).

Tumor stage data were available in Profile for only a subset of breast, colorectal, lung, and prostate tumors (Table 1). To provide a consistent form of context for our mutation prevalences across all anatomic sites studied, for each site we also present United States national data from the Surveillance, Epidemiology, and End Results Program (SEER) on the proportion of patients diagnosed with regional or distant disease (Figures 1 and 2; Supplementary Table 3), though no SEER data were available for non-melanoma skin cancer [13]. Though based on a different set of tumors than the Profile data, the SEER data provide an indirect comparison between our observed prevalences of primary carcinomas with a *CDH1* or *CTNNB1* mutation and proportions of cases diagnosed with some evidence of cancer cell detachment from the primary tumor.

**Table 1:** Tumor stage distributions by *CDH1* and *CTNNB1* mutation status for Profile breast, colorectal, lung, and prostate cancer patients with primary carcinoma specimens and available information on tumor stage

				Tumor Stage			
Gene	Tumor Site	# Mutations	*N*	I	II	III	IV
*CDH1*							
	Breast	>= 1	22	5	6	5	6
		0	154	40	43	19	52
	Colorectal	>= 1	4	1	0	3	0
		0	223	2	37	93	91
	Lung	>= 1	10	1	0	4	5
		0	332	48	47	111	126
	Prostate	>= 1	0	0	0	0	0
		0	56	4	23	17	12
	All 4 Sites	>= 1	36	7	6	12	11
		0	765	94	150	240	281
*CTNNB1*							
	Breast	>= 1	2	0	1	0	1
		0	399	138	126	56	79
	Colorectal	>= 1	8	0	3	3	2
		0	314	5	46	140	123
	Lung	>= 1	11	5	2	2	2
		0	465	68	70	164	163
	Prostate	>= 1	1	0	1	0	0
		0	89	4	28	31	26
	All 4 Sites	>= 1	22	5	7	5	5
		0	1,267	215	270	391	391

*CTNNB1* was assessed in both OncoMap and OncoPanel platforms. *CDH1* was assessed in OncoPanel only. In both platforms, most patients assessed did not have information on tumor stage available.

For *CDH1*, the prevalence across all tumor sites of primary carcinomas having at least one mutation was 4.1% (Supplementary Table 1). By anatomic site, the prevalence was 4.0% or less for 10 tumor sites (Figure 1). The anatomic sites with mutation prevalences greater than 4.0% were non-melanoma skin (10.7%), breast (12.4%), and stomach (16.7%). Notably, skin and stomach had the two smallest sample sizes of all anatomic sites. Across all sites, most subjects with at least one mutation had exactly one *CDH1* mutation (Figure 1). 74% of *CDH1* mutations were nucleotide substitutions (Supplementary Table 1). At least half of the mutations were nucleotide substitutions for every tumor site. In 9 sites, nucleotide substitutions accounted for 90% or more of mutations. The only cases where insertion and deletion mutations were notably common was in colorectal cancer, in which 23% of mutations were deletions, and breast cancer, in which insertions and deletions each accounted for 24% of mutations. For every site, the SEER proportion of patients diagnosed with regional or distant disease was larger than our observed prevalence of carcinomas with at least one *CDH1* mutation (Figure 1).

For *CTNNB1*, the prevalence across all tumor sites of primary carcinomas having at least one mutation was 3.6% (Supplementary Table 2). By tumor site, the mutation prevalence was 5.0% or less for 12 tumor sites (Figure 2). Endometrial cancer had a mutation prevalence of 15.6%. For all sites, most subjects with at least one mutation had exactly one *CTNNB1* mutation, and 97% of *CTNNB1* mutations were nucleotide substitutions (Supplementary Table 2). Nucleotide substitutions accounted for at least 80% of mutations for every site. Deletions accounted for 20% of breast cancer mutations and 17% of esophageal mutations, but the numbers of observed mutations in these sites were small. As with *CDH1*, for every site the SEER proportion of patients diagnosed with regional or distant disease exceeded our observed prevalence of primary carcinomas with at least one *CTNNB1* mutation (Figure 2).

Sufficient Profile tumor stage data to conduct analysis was only available for breast, colorectal, lung, and prostate cancer. Tumor stage was available for 401 breast cancer patients (33% of total available sample), 322 (34%) colorectal cancer patients, 476 (29%) lung cancer patients, and 90 (16%) prostate cancer patients (Table 1). Among those with tumor stage available for these four anatomic sites, the proportion diagnosed with regional and distant disease, respectively, was 14% and 20% for breast cancer; 44% and 39% for colorectal cancer; 35% and 35% for lung cancer; and 34% and 29% for prostate cancer. Across these four sites, of 544 patients assessed for *CDH1* and diagnosed with regional or distant disease, 521 (96%) had no observed *CDH1* mutations. Of 792 patients from these four sites who were assessed for *CTNNB1* and diagnosed with regional or distant disease, 782 (99%) had no observed *CTNNB1* mutations.

**Figure 3:**
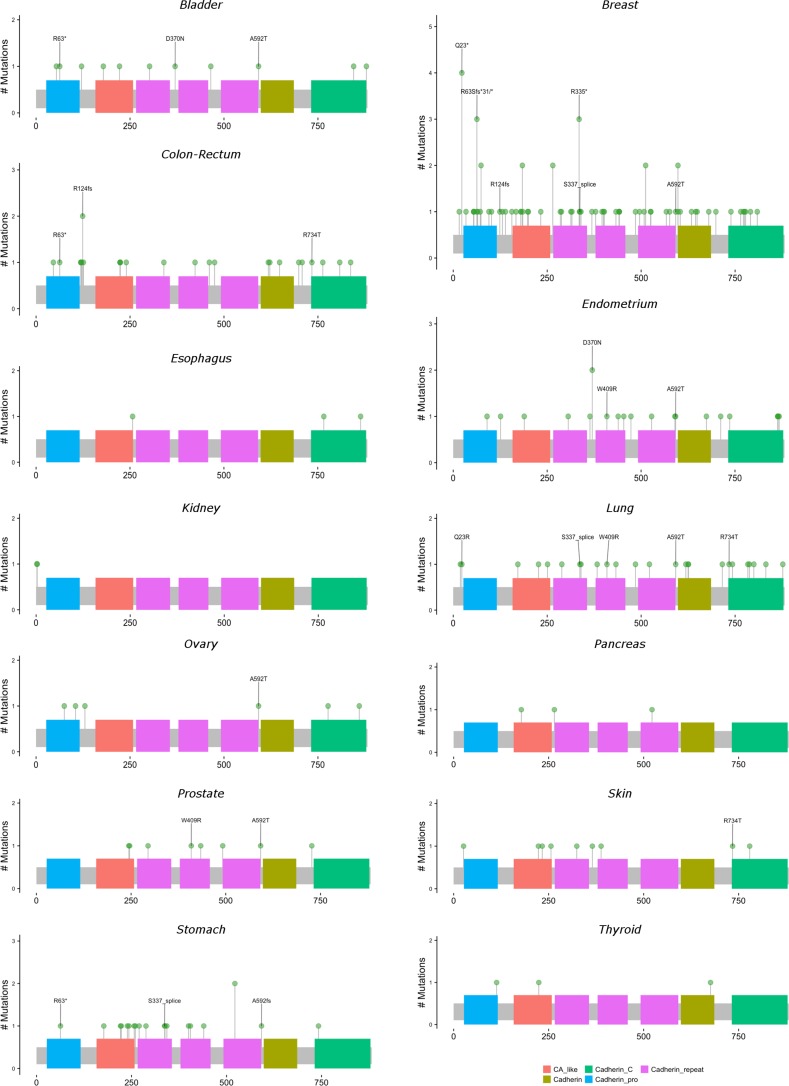
Locations and frequencies of primary carcinoma *CDH1* mutations by anatomic site Excludes observed mutations with missing information on amino acid position. Locations with at least 3 observed mutations across all anatomic sites are labeled.

**Figure 4:**
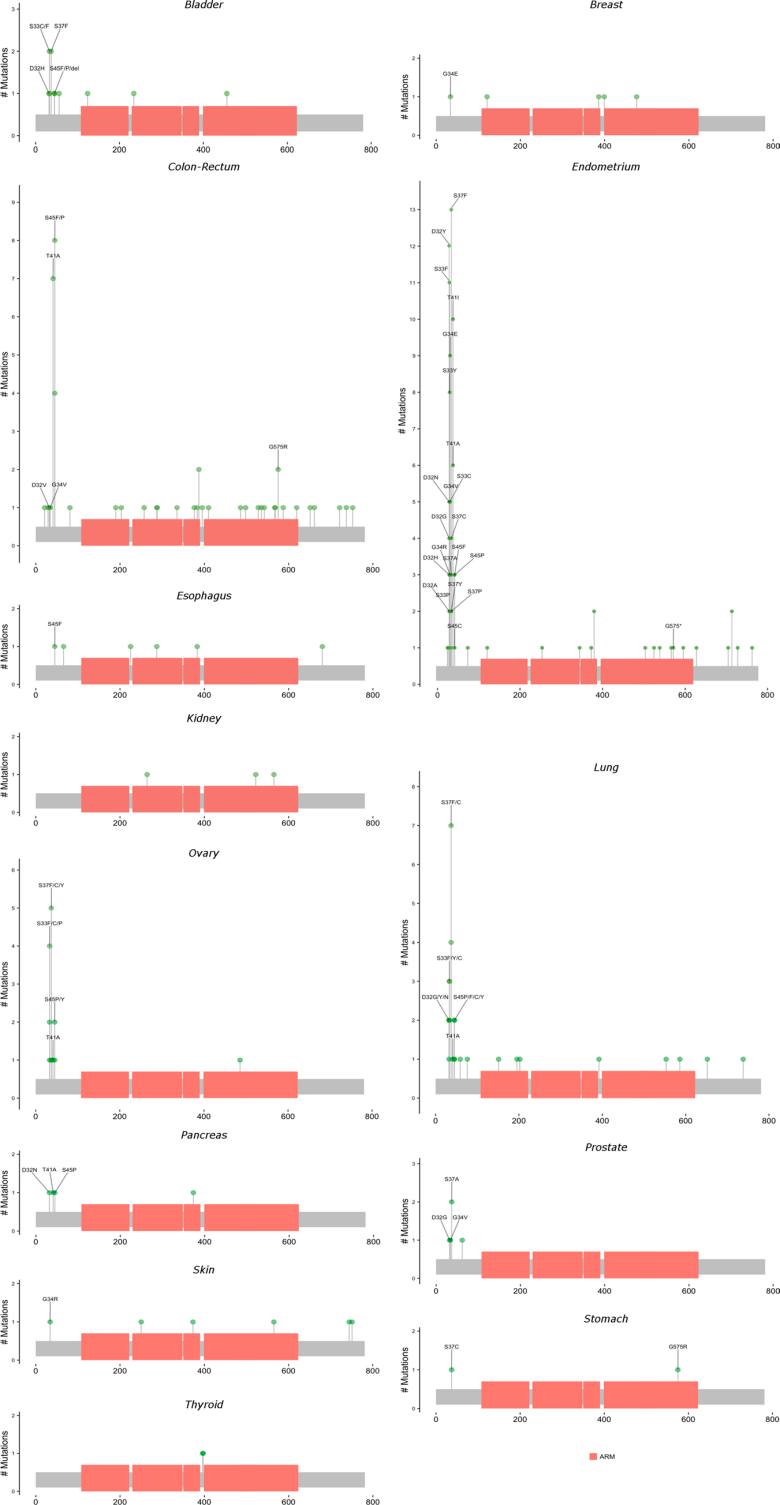
Locations and frequencies of primary carcinoma *CTNNB1* mutations by anatomic site Excludes observed mutations with missing information on amino acid position. Locations with at least 3 observed mutations across all anatomic sites are labeled.

Figures 3 and 4 present lollipop diagrams for *CDH1* and *CTNNB1*, respectively, that map the location by anatomic site of each mutation within the gene. Observed mutations for which amino acid position was not available were excluded from lollipop diagrams. Figures 3 and 4 label amino acid locations at which 3 or more mutations were observed for the gene across all anatomic sites. Further information on each observed mutation is provided in the Supplementary Table 7. For both genes, mutations were observed throughout the length of the genetic sequence. *CDH1* mutations were spread relatively evenly throughout its sequence, while *CTNNB1* mutations were concentrated near the 5′ region of the gene.

All preceding analyses assumed that ambiguous genotyping results were negative. We conducted a sensitivity analysis in which ambiguous genotyping results were treated as positive mutations; this sensitivity analysis could only be performed for *CTNNB1* (see Materials and Methods). In the sensitivity analysis, the mutation prevalence of primary carcinomas with at least one *CTNNB1* mutation rose to between 5–10% for 8 anatomic sites and between 10–15% for 3 sites (Supplementary Table 4). The sites with mutation prevalences greater than 15% in this sensitivity analysis were stomach (18.0%) and endometrial cancer (21.7%). These “maximum” *CTNNB1* mutation prevalences remained lower for every site than the SEER proportion of cases diagnosed with regional or distant disease (Supplementary Tables 3 and 4).

**Figure 5:**
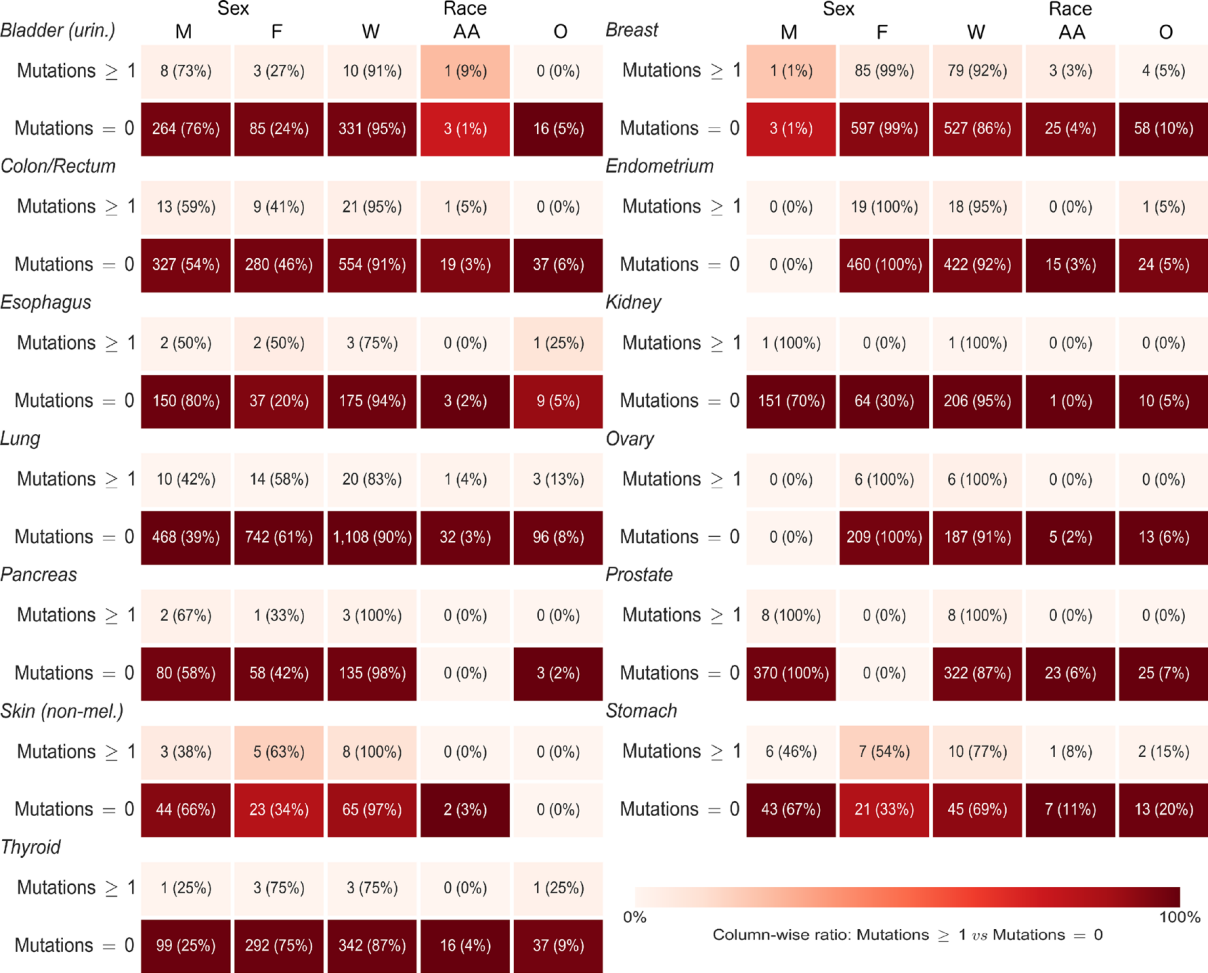
Sex and race distributions by anatomic site and primary carcinoma *CDH1* mutation status (any or no mutations) Numbers in each box are frequency and (demographic variable row percentage). Shading is based on column percentage and normalized by anatomic site. M = Male, F = Female, W = White, AA = African American, O = Other Race.

**Figure 6:**
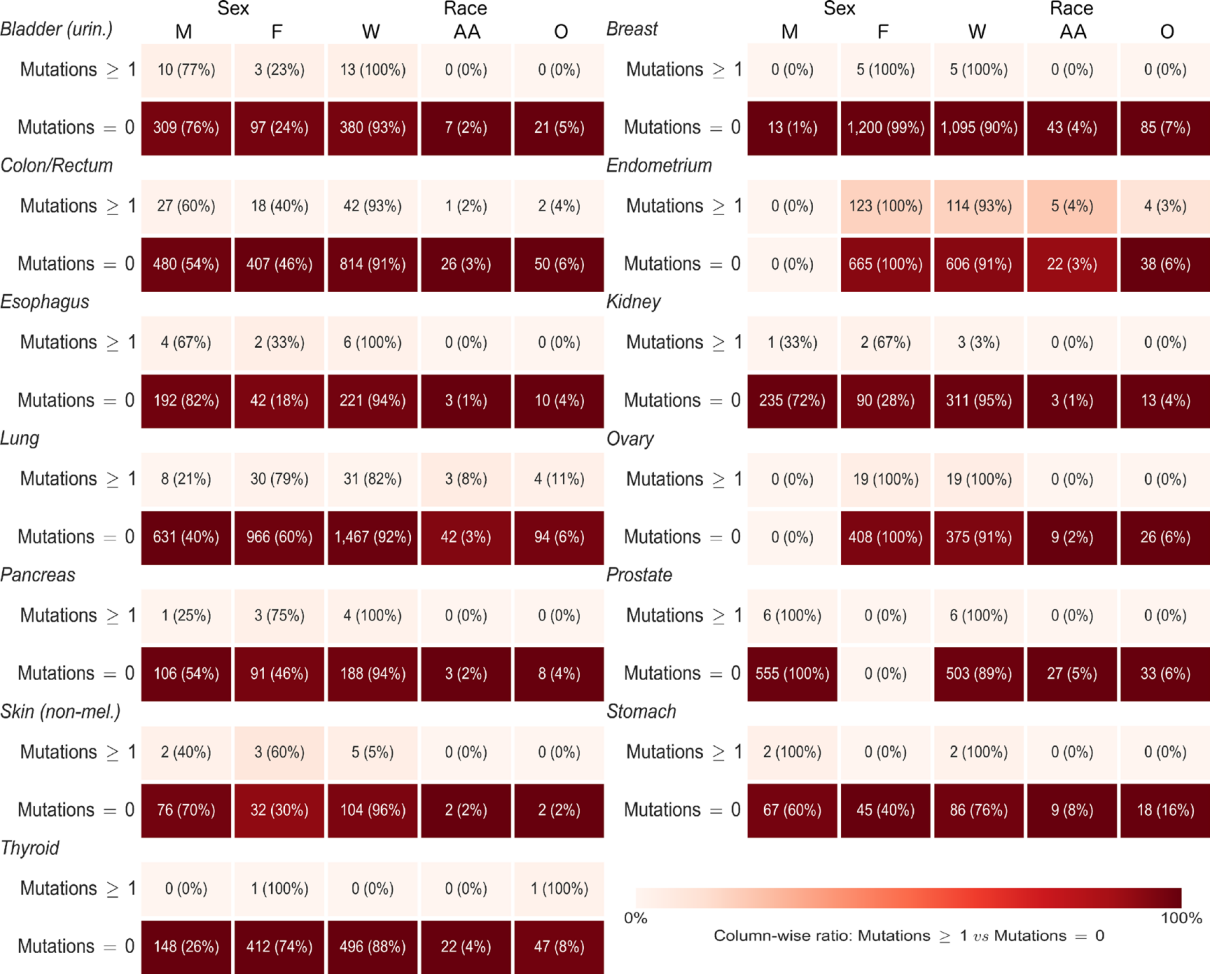
Sex and race distributions by anatomic site and primary carcinoma *CTNNB1* mutation status (any or no mutations) Numbers in each box are frequency and (demographic variable row percentage). Shading is based on column percentage and normalized by anatomic site. M = Male, F = Female, W = White, AA = African American, O = Other Race.

For each gene, we ran cross-tabulations by anatomic site between distributions of mutation status (any or no mutations) and sex, as well as cross-tabulations between mutation status and race (Figure 5 and Supplementary Table 5 for *CDH1*; Figure 6 and Supplementary Table 6 for *CTNNB1*). Among lung cancer patients, women had a higher frequency of *CTNNB1* mutations than men (3% women versus 1% men, *p* = 0.03). We did not observe any other statistically-significant differences in mutation status between women and men, or any differences between African Americans and Whites, for either gene at any anatomic site.

## DISCUSSION

Using thousands of primary carcinomas including the most common forms of cancer, we examined somatic mutations in *CDH1* and *CTNNB1* to assess whether the prevalence of carcinomas with mutations was low, consistent with cancer cell detachment from primary tumors being induced primarily by epigenetic rather than genetic events. We found that, for each gene, over 95% of primary carcinomas across anatomic sites had no *CDH1* or *CTNNB1* mutations, as well as low prevalences of carcinomas with at least one mutation for the large majority of individual sites. Compared to the other anatomic sites, and taking available sample sizes into account, mutations were more prevalent for *CDH1* in breast cancer and for *CTNNB1* in endometrial cancer, though still not a large proportion of carcinomas at these sites.

We hypothesize that if mutations in genes related to EMT, such as *CDH1* or *CTNNB1*, cause a substantial number of cancer cells to detach from a primary tumor, many or most patients with regional or distant disease would have mutations in EMT genes. Limited stage information was available in Profile for breast, colorectal, lung, and prostate cancer, and was essentially not available for the other anatomic sites. Nevertheless, for the four sites with appreciable stage data, we found that the vast majority of subjects with regional or distant disease did not have a primary tumor mutation in either *CDH1* or *CTNNB1* (Table 1). This suggested that mutations in these genes could not explain a large proportion of the cancer cell detachment observed in these cases. Since all the tumors were carcinomas, it is reasonable to think that reduced *CDH1* expression played a role in detachment of cancer cells for patients diagnosed with regional or distant disease, given that *CDH1* is an important epithelial cell-cell adhesion molecule [3]. The low *CDH1* mutation prevalence suggests that, even if *CDH1* mutations contribute to detachment, some mechanism other than *CDH1* mutations was likely responsible for any downregulation of *CDH1* expression for the vast majority of Profile subjects with confirmed diagnoses of regional or distant disease.

Given the limited availability of stage information in our dataset, further indirect context can be gleaned by comparing our prevalences of primary carcinomas with at least one mutation to United States national tumor stage distributions by anatomic site. Comparing SEER stage distribution numbers to our results requires caution because SEER samples are geographically-representative and include all diagnoses for that anatomic site, such as *in situ* tumors or sarcomas. Nevertheless, across sites, the SEER distributions broadly resemble our available Profile stage data in that the proportions of patients displaying evidence of detachment at diagnosis tend to be much greater than our observed *CDH1* or *CTNNB1* mutation prevalences in primary carcinomas.

Furthermore, this discrepancy between observed prevalence of primary carcinomas with a mutation and proportion of patients diagnosed with regional or distant disease suggests that mutations in these genes can at best explain a limited proportion of detachment. For example, we found that 3.5% of colorectal tumors and 1.9% of lung tumors had at least one *CDH1* mutation (Figure 1). However, among Profile patients with tumor stage data available, 187 of 227 (82%) colorectal cancer patients, and 246 of 342 (72%) lung cancer patients, had regional or distant disease (Table 1). In SEER, 56% of colorectal cancer patients, and 79% of lung cancer patients, had regional or distant disease [13]. In the Profile data, of 187 colorectal cancer cases and 246 lung cancer cases evaluated for *CDH1* and with confirmed regional or distant disease, only 3 colorectal cancer cases and 9 lung cancer cases had at least one *CDH1* mutation. This suggests that *CDH1* mutations could explain detachment of cancer cells from the primary tumor in, at most, roughly 1 in 62 (2%) colorectal cancer cases, and 1 in 27 (4%) lung cancer cases, with observed detachment. Indeed, for every anatomic site and for each gene, the mutation prevalence was lower (usually much lower) than the SEER proportion of patients diagnosed with regional or distant disease (Figures 1 and 2; Supplementary Table 3).

In addition, we observed no clear relationship between anatomic sites that had the most mutations and those in which tumors are most likely to metastasize. If mutations induce cancer cells to detach from the primary tumor, then one would expect the prevalence of primary carcinomas with a *CDH1* or *CTNNB1* mutation to be greatest for anatomic sites that are most likely to metastasize. In our data, no such pattern was evident. Most sites from which tumors are most likely to metastasize—colorectal, esophageal, lung, ovarian, and pancreatic cancer—had low prevalences of both *CDH1* and *CTNNB1* mutations. The only site with a notably higher *CTNNB1* mutation prevalence than other sites, endometrial cancer, is not a form of cancer that is especially likely to metastasize, relative to other sites.

Our analysis had several strengths. First, our large sample size provided an informative assessment of the possible role of EMT gene mutations in detachment of cancer cells from primary carcinomas. Most previous studies have generally examined one or two anatomic sites and used much smaller sample sizes [7–10]. We examined *CDH1* and *CTNNB1* mutations in a wide variety of anatomic sites that collectively account for most cancer cases. Since EMT is thought to be a general mechanism of metastasis, evaluating carcinomas originating in many different parts of the body showed whether the results were consistent across sites.

Second, the analysis was restricted to primary carcinomas. EMT-related detachment would be due primarily to decreased expression of epithelial markers, especially *CDH1*, making tumors arising from epithelial cells the most informative evidence regarding any potential role of mutations in triggering detachment. A large recent study of mutations in metastatic tumors also reported low *CTNNB1* mutation prevalences for most forms of cancer studied [11]. However, those results mixed primary tumor and metastasis specimens, and not all tumor sites were restricted to a single class of tumors such as carcinomas. Mutations in metastasis specimens may be difficult to interpret in terms of detachment because of the possibility that the mutation occurred after detachment. In contrast, our analysis included only primary tumor specimens, and the restriction to carcinomas provided the most plausible scenario in which detachment would involve low cancer cell expression of *CDH1*, given that *CDH1* is a critical epithelial cell-cell adhesion molecule [3]. Moreover, metastasis typically involves many cancer cells detaching from a primary tumor. Any mutation that could induce detachment would likely be present in a substantial proportion of primary tumor cancer cells, rather than occurring only in cells that detach. The low prevalence of *CDH1* or *CTNNB1* mutations in primary carcinomas suggested that mutations in these genes likely did not play much of a role in any metastatic disease that developed from these tumors.

Third, for *CTNNB1*, we performed a sensitivity analysis to evaluate the maximum effect of mutation misclassification due to ambiguous genotyping results. The mutation prevalences remained low even when we changed our analytic assumption that all ambiguous genotyping results were negative to an assumption that all ambiguous results were positive. For example, in the sensitivity analysis, the *CTNNB1* mutation prevalence for colorectal cancer increased from 4.8% to 10.8%, and for lung cancer increased from 2.3% to 8.8%. This suggested that mutation prevalences are too low to explain many clinical observations of cancer cell detachment from a primary carcinoma even if all ambiguous genotyping results were classed as positive for a *CTNNB1* mutation.

The analysis had several limitations. Having limited tumor stage data prevented us from exploring more deeply the correlation between EMT gene mutation status and evidence of detachment. *CDH1* was not evaluated in all of the eligible primary carcinomas, reducing the sample size for that gene compared to *CTNNB1*. Since Profile is not a population-based study, the results may not reflect what would be observed in a population clearly defined by time and place. Lacking matched normal tissue specimens, Profile cannot definitively classify a given mutation as somatic or germline, which required that mutations be interpreted as “likely somatic” and added some ambiguity to the interpretation of the results. Further, our results apply to all carcinomas at a given anatomic site. The role of *CDH1* or *CTNNB1* mutations in generating metastases could potentially vary by carcinoma subtypes within an anatomic site, but such a finer-grained consideration was beyond the scope of the present analysis.

An additional limitation was that the selection criteria for tumors included in the Profile study varied by anatomic site. For example, unlike most anatomic sites in Profile, breast tumors were only included if the patient displayed evidence of metastatic disease, either at diagnosis or post-diagnosis. Thus, it is possible that our observed prevalence of primary breast carcinomas with a *CDH1* mutation, which was higher than the prevalences at most other sites, could be inflated relative to what one would find in a population-based sample. Nonetheless, our mutation prevalence estimates for breast cancer cases remained low.

A final limitation was the lack of data on additional genes implicated in EMT. The mechanism begins with increased cellular expression levels of EMT-inducing transcription factors such as *SNAI1*, *SNAI2*, *ZEB1*, *ZEB2*, and *TWIST1*, which leads to decreased expression of epithelial markers and increased expression of mesenchymal markers [3]. It would have been informative to examine mutation prevalences for additional EMT genes, but none were assessed in Profile. While our results generally support mutations having a minimal effect on cancer cell detachment from primary carcinomas, it is possible that mutations in EMT genes not examined here do play a larger role in metastasis.

In thousands of primary carcinomas, we found that the prevalence of tumors with *CDH1* or *CTNNB1* mutations appear to be too low to explain most clinically-observed cases of cancer cell detachment from the primary tumor (i.e. regional or distant tumor spread). Our results support the hypothesis that somatic mutations in *CDH1* or *CTNNB1* are not a major contributor to cancer cell detachment, and thus play a limited role in the etiology of tumor metastasis.

## MATERIALS AND METHODS

### Study population

Subjects were drawn from the Profile study of cancer genomics at DFCI [12]. Since 2011, most DFCI cancer patients have been offered the opportunity to enroll in Profile, which performs genomic sequencing of clinically-acquired neoplastic tissue specimens. The Institutional Review Board at DFCI approved the protocol. All patients provided informed consent to be included in this research.

Profile included tissue specimens from patients with any cancer diagnosis, tumor stage, and type of tissue specimen available (primary tumor, metastasis, recurrence). The present analysis was restricted to carcinoma primary tumor specimens from anatomic sites for which at least 100 primary tumors were successfully sequenced between study inception and June 2016. These sites were urinary bladder, breast, colon/rectum, endometrium, esophagus, kidney, lung, ovary, pancreas, prostate, skin (non-melanoma), stomach, and thyroid. Included carcinoma diagnoses by anatomic site and genotyping platform are listed in the Supplementary Materials. We excluded well-differentiated neuroendocrine (carcinoid) tumors and *in situ* tumors.

Clinical data on race and sex were available for most patients. Tumor stage information was available for a limited number of subjects.

### Tumor genotype data

From study inception through September 2013, sequencing was performed using the OncoMap platform [14]. Starting in June 2013 and continuing through the present, patients were sequenced using the OncoPanel platform [15–17]. Subjects enrolled from June 2013 to September 2013 were sequenced on one of the two platforms but not both, except for 3 patients who were evaluated using both platforms. Of the 3 patients evaluated using both platforms, only 1 met the inclusion criteria (a lung adenocarcinoma patient).

OncoMap consisted of mass spectrometric genotyping of 471 mutations across 41 genes. OncoPanel consisted of hybrid-capture, targeted massively parallel sequencing of approximately 300 genes. OncoMap evaluated hotspot mutations while OncoPanel evaluated exon regions of the genes of interest. For both *CDH1* and *CTNNB1*, neither introns nor 5’ untranslated regions were evaluated. Sequencing was performed only on tumors and not on matched normal tissue. Bioinformatic analysis attempted to filter out likely germline mutations so that observed mutations were restricted to those that were likely somatic, but we cannot be certain that a given mutation was germline or somatic.

Across all anatomic sites and types of tissue specimens, OncoMap genotyping was performed on 4,941 patients. Through June 2016, OncoPanel sequencing was attempted on 12,405 patients. Both preceding totals for OncoMap and OncoPanel include the 3 patients who were evaluated using both platforms. The OncoPanel assay failed for 187 patients (1.5%), yielding 12,218 patients successfully sequenced using this platform.

*CTNNB1* was evaluated in both platforms. *CDH1* was evaluated in OncoPanel only. In OncoMap, roughly 33 hotspots per gene were evaluated, with an explicit result of Present (positive mutation), Absent (no mutation), or No Call (ambiguous) recorded for each hotspot for each gene. In OncoPanel, only positive mutations were recorded, meaning that Absent and No Call observations were not distinguished from each other.

### Statistical analysis

For each anatomic site among all included cases in OncoPanel, the prevalence of primary tumors with at least one *CDH1* mutation was calculated as the number of primary tumors with at least one positive *CDH1* mutation divided by the number of primary tumors evaluated. The prevalence of tumors with at least one *CTNNB1* mutation for each tumor site was calculated analogously for OncoMap and OncoPanel patients combined. After combining *CTNNB1* OncoMap and OncoPanel data for lung cancer patients, we excluded OncoMap data for the single included patient who was evaluated using both platforms to avoid double-counting.

For each gene and platform, the main calculations of prevalence of carcinomas with a mutation assumed negative results in all situations other than unambiguous, explicit positive mutations. This meant that OncoMap “No Call” observations were treated as negative, and in OncoPanel an absence of positive mutations was treated as no mutations. This provided the minimum prevalence of primary carcinomas with at least one mutation in a given gene based on the data.

As a sensitivity analysis for *CTNNB1*, we calculated the prevalence if all “No Call” observations from OncoMap were treated as positive results, and further assumed that the proportion of ambiguous results in OncoPanel was the same as was observed in OncoMap. This provided an estimate of the maximum possible prevalence based on the data. To do this, we calculated the proportion of OncoMap cases with at least one “No Call” observation and no positive mutations, then applied this proportion to the combined total number of OncoMap and OncoPanel cases for the anatomic site to add an estimated number of “possible positive” patients to those observed to have a positive result. This sensitivity analysis could not be performed for *CDH1* due to the lack of OncoMap data.

For a given gene and anatomic site, after stratifying patients by mutation status (any or no mutations), we examined distributions of sex (male or female) and race (White, African American, or other) within each stratum. Due to a large proportion of missing data on Hispanic ethnicity status, the race variable was not coded with a separate category for Hispanics, nor did we cross-tabulate race and Hispanic ethnicity, but no participant was excluded based on race or ethnicity. Fisher’s exact tests were used to evaluate associations between mutation status and sex as well as, for African Americans and Whites, associations between mutation status and race. *P*-values less than 0.05 were considered statistically significant. When tumor stage was available for a substantial proportion of patients for a given anatomic site, we also examined the stage distribution by mutation status.

All statistical analyses were performed using SAS 9.4 (SAS Institute, Cary, NC). Stacked bar charts were generated using Windows Excel 2016 (Microsoft, Redmond, WA). Heat maps and lollipop diagrams were generated using a combination of Python 2.7 (extensions/libraries: Tulip 4.10; SciPy 0.18.1; StatsModels 0.6.1; NumPy 1.11.2) (Python Software Foundation, Beaverton, OR) and R 3.3.2 (extensions/libraries: HeatMapLY 0.60; MafTools 3.4) (R Foundation for Statistical Computing, Vienna, Austria).

### Human rights and informed consent

All procedures performed in studies involving human participants were in accordance with the ethical standards of the institutional and/or national research committee and with the 1964 Helsinki declaration and its later amendments or comparable ethical standards. Informed consent was obtained from all individual participants included in the study.

## SUPPLEMENTARY MATERIALS TABLES





## Abbreviations

*CDH1*: Cadherin-1, E-cadherin (gene)
*CTNNB1*: Catenin beta-1, beta-catenin (gene)
DFCI: Dana-Farber Cancer Institute
EMT: Epithelial-mesenchymal transition
OncDRS: Oncology Data Retrieval System
SEER: Surveillance, Epidemiology, and End Results Program
*SNAI1*: Snail family zinc finger 1 (gene)
*SNAI2*: Snail family zinc finger 2 (gene)
*TWIST1*: Twist Basic Helix-Loop-Helix Transcription Factor 1 (gene)
*ZEB1*: Zinc Finger E-Box Binding Homeobox 1 (gene)
*ZEB2*: Zinc Finger E-Box Binding Homeobox 2 (gene)
