# Supplementary material for: Somatic mutations in CDH1 and CTNNB1 in primary carcinomas at 13 anatomic sites
Source: Oncotarget. 2017 Sep 21;8(49):85680–91. doi: 10.18632/oncotarget.21115 (PMC5689640; doi:10.18632/oncotarget.21115)
Supplement: Supplementary file 1 [file oncotarget-08-85680-s001.pdf]

Somatic mutations in *CDH1* and *CTNNB1* in primary carcinomas at 13 anatomic sites

SUPPLEMENTARY MATERIALS

Included carcinoma diagnosis names by anatomic site and genotyping platform

Bladder cancer

OncoMap (Site: Urinary Bladder)

Adenocarcinoma
Carcinoma
Papillary carcinoma
Small cell carcinoma
Squamous cell carcinoma
Transitional cell carcinoma
Urothelial neoplasm

OncoPanel (Site: BLADDER)

Adenocarcinoma, NOS
Bladder Adenocarcinoma
Bladder Squamous Cell Carcinoma
Bladder Urothelial Carcinoma
Plasmacytoid/Signet Ring Cell Bladder Carcinoma
Small Cell Bladder Cancer
Upper Tract Urothelial Carcinoma
Urachal Carcinoma

Breast cancer

OncoMap (Site: Breast)

Adenocarcinoma
Carcinoma
Ductal-endocrine carcinoma
Squamous cell carcinoma
adenoid cystic carcinoma
breast carcinoma

OncoPanel (Site: BREAST)

Adenoid Cystic Breast Cancer
Breast Invasive Carcinosarcoma, NOS
Breast Invasive Ductal Carcinoma
Breast Invasive Lobular Carcinoma
Breast Invasive Mixed Mucinous Carcinoma
Breast Mixed Ductal and Lobular Carcinoma
Carcinoma with Chondroid Metaplasia
Epithelial-Myoepithelial Carcinoma
Invasive Breast Carcinoma
Metaplastic Breast Cancer
Solid Papillary Carcinoma of the Breast

Colorectal cancer

OncoMap (Site: Colon/Rectum)

Adenocarcinoma
Carcinoma
Squamous cell carcinoma

OncoPanel (Site: BOWEL)

Colon Adenocarcinoma
Colorectal Adenocarcinoma
High-Grade Neuroendocrine Carcinoma of the Colon and Rectum
Mucinous Adenocarcinoma of the Colon and Rectum
Rectal Adenocarcinoma

Endometrial cancer

OncoMap (Site: Endometrium)

Adenocarcinoma
Carcinoma
Clear cell carcinoma
Small cell carcinoma
Endometrial

OncoPanel (Site: UTERUS)

Endometrial Carcinoma
Uterine Clear Cell Carcinoma
Uterine Dedifferentiated Carcinoma
Uterine Endometrioid Carcinoma
Uterine Mixed Endometrial Carcinoma
Uterine Neuroendocrine Carcinoma
Uterine Serous Carcinoma/Uterine Papillary Serous Carcinoma
Uterine Undifferentiated Carcinoma

Esophageal cancer

OncoMap (Site: Esophagus)

Adenocarcinoma
Carcinoma
Squamous cell carcinoma

OncoPanel (Site: STOMACH)

Adenocarcinoma of the Gastroesophageal Junction
Esophageal Adenocarcinoma
Esophageal Poorly Differentiated Carcinoma
Esophageal Squamous Cell Carcinoma
Esophagogastric Adenocarcinoma

Kidney cancer

OncoMap (Site: Kidney)

Carcinoma
Papillary carcinoma
Renal cell carcinoma
clear cell renal carcinoma

OncoPanel (Site: KIDNEY)

Chromophobe Renal Cell Carcinoma
Collecting Duct Renal Cell Carcinoma
Papillary Renal Cell Carcinoma
Renal Cell Carcinoma
Renal Clear Cell Carcinoma
Renal Non-Clear Cell Carcinoma
Renal Small Cell Carcinoma
Sarcomatoid Renal Cell Carcinoma
Translocation-Associated Renal Cell Carcinoma
Unclassified Renal Cell Carcinoma

Lung cancer

OncoMap (Site: Lung)

Adenocarcinoma
Carcinoma
Neuroendocrine carcinoma
Neuroendocrine tumor
Non-small cell carcinoma
Small cell carcinoma
Squamous cell carcinoma

OncoPanel (Site: LUNG)

Adenoid Cystic Carcinoma of the Lung
Combined Small Cell Lung Carcinoma
Giant Cell Carcinoma of the Lung
Large Cell Lung Carcinoma
Large Cell Neuroendocrine Carcinoma
Lung Adenocarcinoma
Lung Adenosquamous Carcinoma
Lung Squamous Cell Carcinoma
Myoepithelial Carcinoma
Neuroendocrine Carcinoma, NOS
Non-Small Cell Lung Cancer
Poorly Differentiated Non-Small Cell Lung Cancer
Sarcomatoid Carcinoma of the Lung
Small Cell Lung Cancer

Ovarian cancer

OncoMap (Site: Ovary)

Adenocarcinoma
Carcinoma
Clear cell carcinoma
Cystadenocarcinoma
Papillary carcinoma
Small cell carcinoma
Transitional cell carcinoma
clear cell ovarian carcinoma

OncoPanel (Site: OVARY)

Clear Cell Ovarian Cancer
Endometrioid Ovarian Cancer
High-Grade Serous Ovarian Cancer
Low-Grade Serous Ovarian Cancer
Mixed Ovarian Carcinoma
Mucinous Ovarian Cancer
Ovarian Seromucinous Carcinoma
Poorly Differentiated Carcinoma, NOS
Serous Ovarian Cancer
Small Cell Carcinoma of the Ovary
Uterine Clear Cell Carcinoma

Pancreatic cancer

OncoMap (Site: Pancreas)

Adenocarcinoma
Carcinoma
Neuroendocrine carcinoma

OncoPanel (Site: PANCREAS)

Acinar Cell Carcinoma of the Pancreas
Adenocarcinoma, NOS
Adenosquamous Carcinoma of the Pancreas
Pancreatic Adenocarcinoma
Poorly Differentiated Carcinoma, NOS

Prostate cancer

OncoMap (Site: Prostate)

Adenocarcinoma
Carcinoma
Neuroendocrine carcinoma

OncoPanel (Site: PROSTATE)

Prostate Adenocarcinoma
Prostate Neuroendocrine Carcinoma
Prostate Sqamous Cell Carcinoma

Skin cancer (non-melanoma)

OncoMap (Site: Skin)

Carcinoma
Merkel Cell Carcinoma
Small cell carcinoma
Squamous cell carcinoma
skin basal cell

OncoPanel (Site: SKIN)

Adenocarcinoma, NOS
Basal Cell Carcinoma
Cutaneous Squamous Cell Carcinoma
Endocrine Mucin Producing Sweat Gland Carcinoma
Merkel Cell Carcinoma
Microcystic Adnexal Carcinoma
Skin Adnexal Carcinoma
Squamous Cell Carcinoma, NOS

Stomach cancer

OncoMap (Site: Stomach)

Adenocarcinoma
Carcinoma
Neuroendocrine carcinoma

OncoPanel (Site: STOMACH)

Adenosquamous Carcinoma of the Stomach
Diffuse Type Stomach Adenocarcinoma
Intestinal Type Stomach Adenocarcinoma
Papillary Stomach Adenocarcinoma
Poorly Differentiated Carcinoma of the Stomach
Signet Ring Cell Carcinoma of the Stomach
Stomach Adenocarcinoma
Tubular Stomach Adenocarcinoma

Thyroid cancer

OncoMap (Site: Thyroid)

Adenocarcinoma
Carcinoma
Papillary carcinoma
follicular thyroid
medullary thyroid
papillary thyroid

OncoPanel (Site: THYROID)

Anaplastic Thyroid Cancer
Follicular Thyroid Cancer
Hurthle Cell Thyroid Cancer
Medullary Thyroid Cancer
Papillary Thyroid Cancer
Poorly Differentiated Thyroid Cancer

Supplementary Table 1: *CDH1* mutations in Profile primary carcinomas

Tumor Site	Mutation Prevalence			Total # Mutations	Mutation Type Distribution[a]		
	# individuals w/ >= 1 mutation (x)	# individuals assessed (y)	Prevalence (%) (x/y)		# (%) Substitution	# (%) Insertion	# (%) Deletion
**Bladder (urinary)**	11	361	3.0	11	10 (91)	1 (9)	0 (0)
**Breast**	86	696	12.4	87	45 (52)	21 (24)	21 (24)
**Colon/Rectum**	22	632	3.5	26	18 (69)	2 (8)	6 (23)
**Endometrium**	19	480	4.0	22	21 (95)	0 (0)	1 (5)
**Esophagus**	4	191	2.1	4	2 (50)	0 (0)	2 (50)
**Kidney**	1	218	0.5	1	1 (100)	0 (0)	0 (0)
**Lung**	24	1,240	1.9	25	25 (100)	0 (0)	0 (0)
**Ovary**	6	215	2.8	6	6 (100)	0 (0)	0 (0)
**Pancreas**	3	143	2.1	3	3 (100)	0 (0)	0 (0)
**Prostate**	8	378	2.1	8	8 (100)	0 (0)	0 (0)
**Skin (non-melanoma)**	8	75	10.7	9	9 (100)	0 (0)	0 (0)
**Stomach**	13	78	16.7	20	16 (80)	2 (10)	2 (10)
**Thyroid**	4	399	1.0	4	4 (100)	0 (0)	0 (0)
**All 13 Sites**	209	5,106	4.1	226	168 (74)	26 (12)	32 (14)

[a]Denominator for mutation type percentages is the total number of mutations.

Supplementary Table 2: *CTNNB1* mutations in Profile primary carcinomas

Tumor Site	Mutation Prevalence			Total # Mutations	Mutation Type Distribution[a]		
	# individuals w/ >= 1 mutation (x)	# individuals assessed (y)	Prevalence (%) (x/y)		# (%) Substitution	# (%) Insertion	# (%) Deletion
**Bladder (urinary)**	13	421	3.1	13	12 (92)	0 (0)	1 (8)
**Breast**	5	1,228	0.4	5	4 (80)	0 (0)	1 (20)
**Colon/Rectum**	45	935	4.8	53	51 (96)	1 (2)	1 (2)
**Endometrium**	123	789	15.6	138	136 (99)	0 (0)	2 (1)
**Esophagus**	6	240	2.5	6	5 (83)	0 (0)	1 (17)
**Kidney**	3	330	0.9	3	3 (100)	0 (0)	0 (0)
**Lung**	38	1,640	2.3	41	40 (98)	1 (2)	0 (0)
**Ovary**	19	429	4.4	19	19 (100)	0 (0)	0 (0)
**Pancreas**	4	203	2.0	4	4 (100)	0 (0)	0 (0)
**Prostate**	6	569	1.1	6	6 (100)	0 (0)	0 (0)
**Skin (non-melanoma)**	5	113	4.4	7	7 (100)	0 (0)	0 (0)
**Stomach**	2	115	1.7	2	2 (100)	0 (0)	0 (0)
**Thyroid**	1	566	0.2	1	1 (100)	0 (0)	0 (0)
**All 13 Sites**	270	7,578	3.6	298	290 (97)	2 (1)	6 (2)

[a]Denominator for mutation type percentages is the total number of mutations.

Supplementary Table 3: Comparison between Profile EMT gene mutation prevalences and SEER proportions of patients with clinically-observed cancer cell detachment

Tumor Site	SEER Stage Distribution[a]			Profile[b]	
	Regional (%)	Distant (%)	Regional + Distant (%)	Prevalence of tumors with >= 1 CDH1 mutation (%)	Prevalence of tumors with >= 1 CTNNB1 mutation (%)
**Bladder (urinary)**	7	4	11	3.0	3.1
**Breast**	31	6	37	12.4	0.4
**Colon/Rectum**	35	21	56	3.5	4.8
**Endometrium**	21	9	30	4.0	15.6
**Esophagus**	31	39	70	2.1	2.5
**Kidney**	16	16	32	0.5	0.9
**Lung**	22	57	79	1.9	2.3
**Ovary**	20	60	80	2.8	4.4
**Pancreas**	29	52	81	2.1	2.0
**Prostate**	12	5	17	2.1	1.1
**Skin (non-melanoma)**		Data not available		10.7	4.4
**Stomach**	28	35	63	16.7	1.7
**Thyroid**	27	4	31	1.0	0.2

[a]Percentages are out of all tumors at the tumor site (in situ, local, regional, distant). Tumors not necessarily restricted to carcinomas. Source: Howlader N, Noone AM, Krapcho M, Miller D, Bishop K, Kosary CL, Yu M, Ruhl J, Tatalovich Z, Mariotto A, Lewis DR, Chen HS, Feuer EJ, Cronin KA (eds). SEER Cancer Statistics Review, 1975–2014, National Cancer Institute. Bethesda, MD, https://seer.cancer.gov/csr/1975_2014/, based on November 2016 SEER data submission, posted to the SEER web site, April 2017.
[b]Prevalence of tumors with at least one mutation for the gene among primary carcinomas.

Supplementary Table 4: Estimated *CTNNB1* mutation prevalence if ambiguous observations assumed to be positive

Tumor Site	# PP[a], Map	# Cases, Map	# Cases, Map + Panel	Estimated PP, Map + Panel	# OP[b], Map + Panel	# (OP+PP), Map + Panel	Estimated Maximum Prevalence (%), Map + Panel
**Bladder (urinary)**	3	60	421	21.05	13	34.05	8.1
**Breast**	45	532	1,228	103.87	5	108.87	8.9
**Colon/Rectum**	18	303	935	55.54	45	100.54	10.8
**Endometrium**	19	309	789	48.51	123	171.51	21.7
**Esophagus**	3	49	240	14.69	6	20.69	8.6
**Kidney**	10	112	330	29.46	3	32.46	9.8
**Lung**	26	401	1,641	106.40	38	144.40	8.8
**Ovary**	20	217	429	39.54	19	58.54	13.6
**Pancreas**	2	60	203	6.77	4	10.77	5.3
**Prostate**	20	192	569	59.27	6	65.27	11.5
**Skin (non-melanoma)**	1	38	113	2.97	5	7.97	7.1
**Stomach**	6	37	115	18.65	2	20.65	18.0
**Thyroid**	12	167	566	40.67	1	41.67	7.4

Calculations assume that the proportion of Possible Positives for a given tumor site is the same in OncoPanel as was observed in OncoMap.
[a]Possible Positives are subjects who had at least one ambiguous *CTNNB1* observation and no positive *CTNNB1* mutations.
[b]Observed Positives are subjects who had at least one positive CTNNB1 mutation.
Map = OncoMap, OP = Observed Positives, Panel = OncoPanel, PP = Possible Positive.

Supplementary Table 5: Patient demographics by *CDH1* mutation status

Tumor Site	# *CDH1* Mutations	N	Sex[a]		Race[a]		
			M (%)	F (%)	W (%)	AA (%)	Other[b] (%)
**Bladder (urinary)**	>= 1	11	8 (73)	3 (27)	10 (91)	1 (9)	0 (0)
	0	350	264 (76)	85 (24)	331 (95)	3 (1)	16 (5)
**Breast**	>= 1	86	1 (1)	85 (99)	79 (92)	3 (3)	4 (5)
	0	610	3 (1)	597 (99)	527 (86)	25 (4)	58 (10)
**Colon/Rectum**	>= 1	22	13 (59)	9 (41)	21 (95)	1 (5)	0 (0)
	0	610	327 (54)	280 (46)	554 (91)	19 (3)	37 (6)
**Endometrium**	>= 1	19	0 (0)	19 (100)	18 (95)	0 (0)	1 (5)
	0	461	0 (0)	460 (100)	422 (92)	15 (3)	24 (5)
**Esophagus**	>= 1	4	2 (50)	2 (50)	3 (75)	0 (0)	1 (25)
	0	187	150 (80)	37 (20)	175 (94)	3 (2)	9 (5)
**Kidney**	>= 1	1	1 (100)	0 (0)	1 (100)	0 (0)	0 (0)
	0	217	151 (70)	64 (30)	206 (95)	1 (0)	10 (5)
**Lung**	>= 1	24	10 (42)	14 (58)	20 (83)	1 (4)	3 (13)
	0	1,216	468 (39)	742 (61)	1,108 (90)	32 (3)	96 (8)
**Ovary**	>= 1	6	0 (0)	6 (100)	6 (100)	0 (0)	0 (0)
	0	209	0 (0)	209 (100)	187 (91)	5 (2)	13 (6)
**Pancreas**	>= 1	3	2 (67)	1 (33)	3 (100)	0 (0)	0 (0)
	0	140	80 (58)	58 (42)	135 (98)	0 (0)	3 (2)
**Prostate**	>=1	8	8 (100)	0 (0)	8 (100)	0 (0)	0 (0)
	0	370	370 (100)	0 (0)	322 (87)	23 (6)	25 (7)
**Skin (non-melanoma)**	>=1	8	3 (38)	5 (63)	8 (100)	0 (0)	0 (0)
	0	67	44 (66)	23 (34)	65 (97)	2 (3)	0 (0)
**Stomach**	>=1	13	6 (46)	7 (54)	10 (77)	1 (8)	2 (15)
	0	65	43 (67)	21 (33)	45 (69)	7 (11)	13 (20)
**Thyroid**	>=1	4	1 (25)	3 (75)	3 (75)	0 (0)	1 (25)
	0	395	99 (25)	292 (75)	342 (87)	16 (4)	37 (9)
**All 13 Sites**	>=1	209	55 (26)	154 (74)	190 (91)	7 (3)	12 (6)
	0	4,897	1,999 (41)	2,898 (59)	4,419 (90)	151 (3)	341 (7)

[a]Denominators of percentages for patient characteristics are the sum of the patients across the characteristic categories, not N. Differences between N and the sum of patients across characteristic categories are missing data.

[b]Includes unknown race.

M = Male, F = Female, W = White, AA = African American.

Supplementary Table 6: Patient demographics by *CTNNB1* mutation status

			Sex[a]		Race[a]		
**Tumor Site**	**# *CTNNB1* Mutations**	**N**	**M (%)**	**F (%)**	**W (%)**	**AA (%)**	**Other[b] (%)**
**Bladder (urinary)**	>= 1	13	10 (77)	3 (23)	13 (100)	0 (0)	0 (0)
	0	408	309 (76)	97 (24)	380 (93)	7 (2)	21 (5)
**Breast**	>= 1	5	0 (0)	5 (100)	5 (100)	0 (0)	0 (0)
	0	1,223	13 (1)	1,200 (99)	1,095 (90)	43 (4)	85 (7)
**Colon/Rectum**	>= 1	45	27 (60)	18 (40)	42 (93)	1 (2)	2 (4)
	0	890	480 (54)	407 (46)	814 (91)	26 (3)	50 (6)
**Endometrium**	>= 1	123	0 (0)	123 (100)	114 (93)	5 (4)	4 (3)
	0	666	0 (0)	665 (100)	606 (91)	22 (3)	38 (6)
**Esophagus**	>= 1	6	4 (67)	2 (33)	6 (100)	0 (0)	0 (0)
	0	234	192 (82)	42 (18)	221 (94)	3 (1)	10 (4)
**Kidney**	>= 1	3	1 (33)	2 (67)	3 (3)	0 (0)	0 (0)
	0	327	235 (72)	90 (28)	311 (95)	3 (1)	13 (4)
**Lung**	>= 1	38	8 (21)	30 (79)	31 (82)	3 (8)	4 (11)
	0	1,603	631 (40)	966 (60)	1,467 (92)	42 (3)	94 (6)
**Ovary**	>= 1	19	0 (0)	19 (100)	19 (100)	0 (0)	0 (0)
	0	410	0 (0)	408 (100)	375 (91)	9 (2)	26 (6)
**Pancreas**	>= 1	4	1 (25)	3 (75)	4 (100)	0 (0)	0 (0)
	0	199	106 (54)	91 (46)	188 (94)	3 (2)	8 (4)
**Prostate**	>= 1	6	6 (100)	0 (0)	6 (100)	0 (0)	0 (0)
	0	563	555 (100)	0 (0)	503 (89)	27 (5)	33 (6)
**Skin (non-melanoma)**	>= 1	5	2 (40)	3 (60)	5 (5)	0 (0)	0 (0)
	0	108	76 (70)	32 (30)	104 (96)	2 (2)	2 (2)
**Stomach**	>= 1	2	2 (100)	0 (0)	2 (100)	0 (0)	0 (0)
	0	113	67 (60)	45 (40)	86 (76)	9 (8)	18 (16)
**Thyroid**	>= 1	1	0 (0)	1 (100)	0 (0)	0 (0)	1 (100)
	0	565	148 (26)	412 (74)	496 (88)	22 (4)	47 (8)
**All 13 Sites**	>= 1	270	61 (23)	209 (77)	250 (93)	9 (3)	11 (4)
	0	7,308	2,812 (39)	4,455 (61)	6,645 (91)	218 (3)	445 (6)

[a]Denominators of percentages for patient characteristics are the sum of the patients across the characteristic categories, not N. Differences between N and the sum of patients across characteristic categories are missing data.
[b]Includes unknown race.
M = Male, F = Female, W = White, AA = African American.

Supplementary Table 7: Tumor site, mutation name and location, and patient demographics for each *CDH1* and *CTNNB1* mutation observed in Profile primary carcinomas. See separate Supplementary Table 7 file (Excel)
